# Sine ventilation in lung injury models: a new perspective for lung protective ventilation

**DOI:** 10.1038/s41598-020-68614-x

**Published:** 2020-07-16

**Authors:** Sashko Spassov, Christin Wenzel, Sara Lozano-Zahonero, Dimona Boycheva, Lea Streicher, Johannes Schmidt, Stefan Schumann

**Affiliations:** Workgroup Clinical Respiratory Physiology, Department of Anesthesiology and Critical Care, Medical Center – University of Freiburg, Faculty of Medicine, University of Freiburg, Hugstetter Str. 55, 79106 Freiburg, Germany

**Keywords:** Physiology, Respiration

## Abstract

Mechanical ventilation is associated with the risk of ventilator induced lung injury. For reducing lung injury in mechanically ventilated patients, the application of small tidal volumes and positive end-expiratory pressures has become clinical standard. Recently, an approach based on linear airway pressure decline and decelerated expiratory flow during expiration implied lung protective capacities. We assumed that ventilation with a smoothed, i.e. sinusoidal airway pressure profile may further improve ventilation efficiency and lung protection. We compared the effects of mechanical ventilation with sinusoidal airway pressure profile (SINE) regarding gas exchange, respiratory system compliance and histology to conventional volume and pressure controlled ventilation (VCV and PCV) and to VCV with flow-controlled expiration (FLEX) in two rat models of lung injury, tween induced surfactant depletion and high tidal volume mechanical ventilation. In both lung injury models ventilation with SINE showed more efficient CO_2_ elimination and blood oxygenation, improved respiratory system compliance and resulted in lower alveolar wall thickness, compared to VCV, PCV and FLEX. Optimization of the airway pressure profile may provide a novel means of lung protective mechanical ventilation.

## Introduction

Although mechanical ventilation is a life-saving therapy in critical care medicine, it is not therapeutic but rather “buys time until a causal therapy becomes effective” (Luciano Gattinoni^1^). Nonetheless, mechanical ventilation is per se associated with the risk of ventilator induced lung injury^2^. Strategies of lung protective mechanical ventilation such as the application of small tidal volumes or positive end-expiratory pressure (PEEP) have become well established in clinical practice. However, they are restricted by the patient’s gas exchange requirement and by the risk of volutrauma, respectively. Therefore, advanced strategies of lung protective mechanical ventilation are needed.

Flow-controlled expiration (FLEX) is a new approach of mechanical ventilation that was realized by combining the inspiration of volume controlled ventilation (VCV) with a linearly decreasing pressure course during expiration instead of almost instant, exponential pressure decay characteristic for conventional ventilation modes^3^. In studies from our group such linearized expiration was associated with lung protective effects^3–7^. This implies that the airway pressure profile of the ventilation mode contains lung protective capacities. In this regard, the most promising profile would be the one with a smooth expiratory flow profile, which may provide additional recruitment during expiration. Consequently, we hypothesised that ventilation with a controlled sinusoidal airway pressure profile (SINE) further improves ventilation efficiency and lung protection.

**Figure 1:**
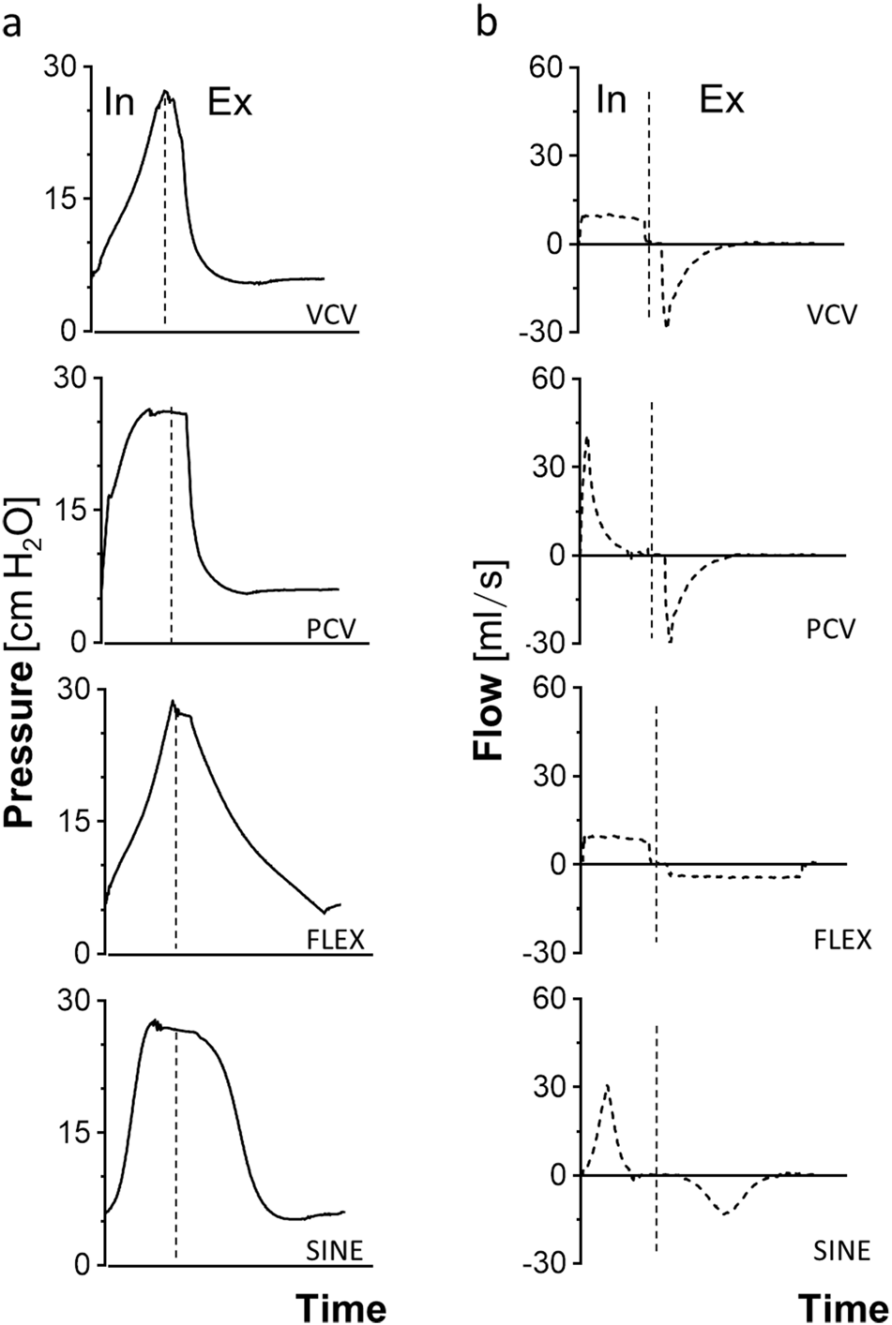
Airway pressure (**a**) and flow profiles (**b**) for volume controlled ventilation (VCV), pressure controlled ventilation (PCV), VCV + flow-controlled expiration (FLEX) and ventilation with sinusoidal airway pressure profile (SINE), respectively. The vertical dashed line separates inspiration (In) from expiration (Ex).

Therefore, we compared the effects of SINE and FLEX to conventional VCV and pressure controlled ventilation (PCV; Fig. 1 corresponding pressure and flow curves) on gas exchange, respiratory system mechanics and lung tissue histology in two separate animal models of lung injury, i.e. tween induced surfactant depletion (Tween) and high tidal volume ventilation (HTVV).

## Methods

### Animal experiments

All animal experiments and related methods were approved and conducted in accordance with the guidelines of the local Animal Care Commission (Ethics Committee University of Freiburg, Germany, permissions No. G-14/116). Housing and animal care procedures took place at the University Medical Center of Freiburg, Germany, and were in compliance with the European directive 2010/63/EU).

Male Sprague Dawley rats with a body weight of 294 ± 30 g (Janvier Labs, Saint-Berthevin, France) were anaesthetised with 100 mg/kg ketamine and 1 mg/kg medetomidine intramuscular, and placed on a heating pad to keep body temperature in the range between 36 and 37 °C. A tracheotomy and tracheal catheterization to facilitate mechanical ventilation, was followed by carotid artery catheterisation for blood pressure monitoring and for drawing blood for blood gas analyses. Before onset of mechanical ventilation, muscular relaxation was induced by intraperitoneal instillation of 1 mg/kg pancuronium. Thereafter, anaesthesia was maintained by continuous administration of ketamine/midazolam/medetomidine (via catheterized femoral vein) and pancuronium as needed. For blood volume maintenance heparinized saline (10 I.E./ml in 0.9% NaCl) was infused intravenously (up to 10 ml/kg/h) to prevent dehydration during ventilation. Heart rate, blood pressure and body temperature were continuously monitored (Hugo Sachs Elektronik-Harvard Apparatus GmbH, March, Germany).

**Figure 2:**
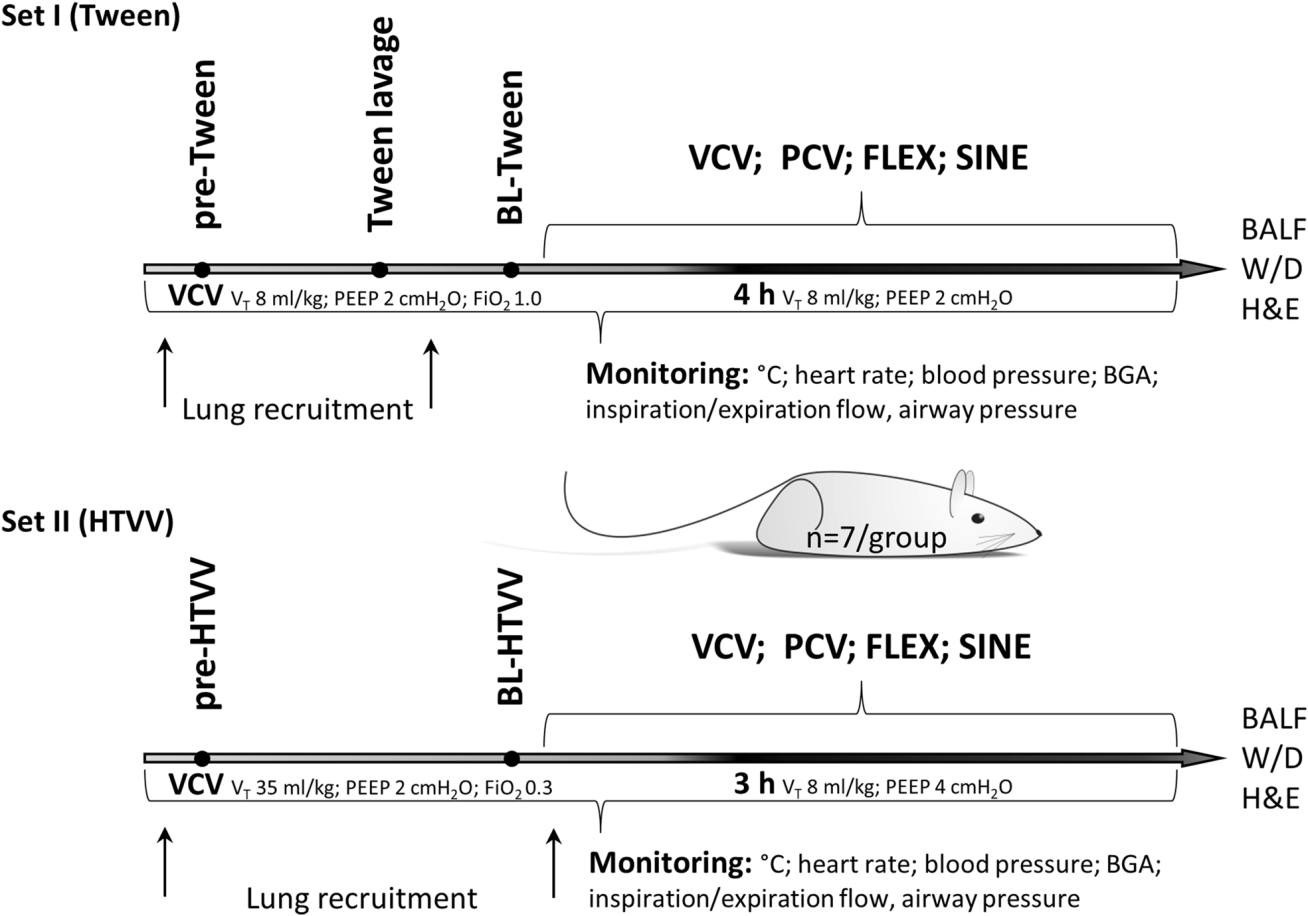
Experimental designs. Set I: surfactant washout model (Tween) and Set II: high tidal volume ventilation (HTVV). At the beginning of the experiments all animals were subjected to volume controlled ventilation until induction of lung injury. Thereafter, in both experimental sets animals were randomized to continue mechanical ventilation with one of the four ventilation modes, volume controlled ventilation (VCV), pressure controlled ventilation (PCV), VCV + flow-controlled expiration (FLEX) and ventilation with a sine pressure course (SINE), (n = 7/group). Experimental and physiological variables were monitored during the entire experiment, *BALF* bronchoalveolar fluid; *BGA* blood gas analysis; *W/D* wet-to-dry ratio.

Animal experiments were run in two subsequent sets (Fig. 2).

### Set I: surfactant washout induced lung injury (Tween)

After surgical preparations, animals were subjected to VCV with a self-developed mechanical ventilator for small animals, which operates with freely designable inspiration and expiration ventilation profiles^8^. Tidal volume (V_T_) was set to 8 ml/kg, FiO_2_ of 1.0, inspiration:expiration ratio of 1:2 and PEEP of 2 cmH_2_O. At the beginning of mechanical ventilation a recruitment manoeuvre was performed. The respiratory rate was set to maintain paCO_2_ at 35 to 45 mmHg. After a stabilization period of 20 min (pre-Tween) lungs were subjected to bronchoalveolar lavage with Tween [3 repetitions with 1 ml 0.1% Tween 80 (Sigma-Aldrich, Taufkirchen, Germany) in 0.9% NaCl]. After the lavage another recruitment manoeuvre was performed and after another thirty minutes arterial blood gases were measured (baseline-Tween). A ratio of arterial oxygen partial pressure and inspiratory oxygen fraction (paO_2_/FiO_2_-ratio) below 150 mmHg, was considered to indicate lung injury. Immediately after induction of lung injury the animals were randomly assigned to mechanical ventilation with VCV, PCV, FLEX or SINE for 4 h (n = 7/group). During the subsequent profile specific ventilation respiratory rate was adjusted if paCO_2_ went outside the range between 35–45 mmHg unless the maximum respiratory rate of the ventilator (95 breaths/min) was achieved. Blood gas analysis was performed 30 min after the beginning of mechanical ventilation (pre-Tween), 20–30 min after the Tween lavage (BL-Tween) and 30, 60, 90, 120, 180 and 240 min thereafter.

### Set II: high tidal volume ventilation (HTVV)

After surgical preparations, the animals were subjected to VCV using a standard mechanical ventilator (Servo-u, Maquet, Göteborg, Schweden) with a V_T_ of 35 ml/kg (FiO_2_ 0.3, inspiration:expiration ratio of 1:2 and PEEP 2 cmH_2_O, respiratory rate set to maintain paCO_2_ at 35–45 mmHg) until lung injury was confirmed by a drop of the paO_2_/FiO_2_-ratio below 350 mmHg (baseline-HTVV). Immediately after lung injury induction the animals were switched to the self-developed small animal ventilator and randomly assigned to mechanical ventilation with VCV, PCV, FLEX or SINE (n = 7/group, V_T_ 8 ml/kg and PEEP 4 cmH_2_O) for 3 h. Lung recruitment manoeuvres were performed at the beginning of the mechanical ventilation and after switching the ventilators. Again respiratory rate was adjusted to maintain paCO_2_ between 35–45 mmHg.

Blood gas analysis was performed 30 min after the beginning of mechanical ventilation (pre-HTVV), at onset of lung injury (BL-HTVV) and 30, 60, 90, 120, 150 and 180 min thereafter.

In both sets of lung injury blood gases were analysed on a regular basis. Airway pressure and flow curves were recorded every 10 min for 2 min. From these curves respiratory system compliance, peak airway pressure (P_peak_) and average (arithmetic mean) airway pressure per respiratory cycle (P_mean_), PEEP, V_T_, average (arithmetic mean) volume per respiratory cycle (V_mean_), and weight adapted minute volume were determined.

At the end of the experiments, rats were exsanguinated. Lung water contents were assessed from the wet-to-dry ratio of the left lung lobe’s weights (measured immediately after excision and after drying for 5 days at 65 °C). Bronchoalveolar lavage fluid (BALF, 3 times repeated washings with 2 ml cold phosphate buffered saline) was collected from the superior and middle right lung lobe. BALF was analysed for pro-inflammatory infiltrates (macrophage and neutrophil cell count), total protein (BCA Protein Assay, ThermoFisher Scientific, Waltham, MA) and macrophage inflammatory protein 2 content (Rat MIP-2 ELISA, LSBio, Seattle, WA).

### Histological analysis

The inferior right lung lobe was fixed with 4% paraformaldehyde, embedded into cryo-mounting medium, frozen and stored at − 80 °C. Cryosections of 6 µm were subjected to haematoxylin and eosin staining. Histological analysis were performed in a blinded manner as previously described^9^. Briefly, alveolar wall thickness, haemorrhage and pro-inflammatory infiltrates were measured/counted in five randomly assigned power fields (identical for all images) in at least 5 images per animal. The thickness of the alveolar walls was documented and analysed using ZEN software (Carl Zeiss Microscopy GmbH, Jena, Germany). The number of measured walls varied between 40 and 105 per image. A lung injury score was calculated as the sum of alveolar wall thickness, haemorrhage and pro-inflammatory infiltrate scores.

For histology analysis lung tissue samples from healthy animals (Control; VCV for 30 min with V_T_ 8 ml/kg; n = 5) were analysed. In the HTVV set additional lung tissue samples from animals with established lung injury (baseline-HTVV; VCV for 2 h with V_T_ 35 ml/kg; n = 5) were analysed.

### Statistics

Data are presented as box plots representing median, upper/lower quartile and bars minimal/maximal values. Box plots present the effects of pre- and BL-injury time points and/or the average effect for the 4 or 3 h mechanical ventilation (for the Tween or HTVV sets, respectively). The time courses of respiratory system compliance are represented as mean±SD. Since equally treated in the corresponding sets, data for pre-Tween, pre-HTVV, baseline-Tween and baseline-HTVV included all animals of the respective set (n = 28). VCV, PCV, FLEX, SINE and controls included n = 7 or n = 5/group, respectively. For comparisons of experimental and physiological variables between the ventilation modes one-way ANOVA, for comparison of the respiratory system compliance between the ventilation modes repeated measures two-way ANOVA were calculated followed by the Tukey post-hoc test (GraphPad Prism 8, GraphPad Software Inc., San Diego, CA, USA or SigmaPlot, Sigmastat software; Systat, Inc., Erkrath, Germany). A *p* value of less than 0.05 was considered significant.

## Results

Data of the four respective groups (VCV, PCV, FLEX and SINE) related to pre-injury and baseline time points were comparable for each respective model (Supplementary Table ST1A and B), and were therefore combined and presented as n = 28 in the following analyses.

### Establishment of lung injury

Before induction of lung injury experimental and physiological variables (e.g. weight, body temperature, respiratory rate, peak pressure, blood haemodynamic and acid–base balance) were within respective reference ranges and comparable within both models of lung injury (Supplementary Table ST2A and B).

**Figure 3:**
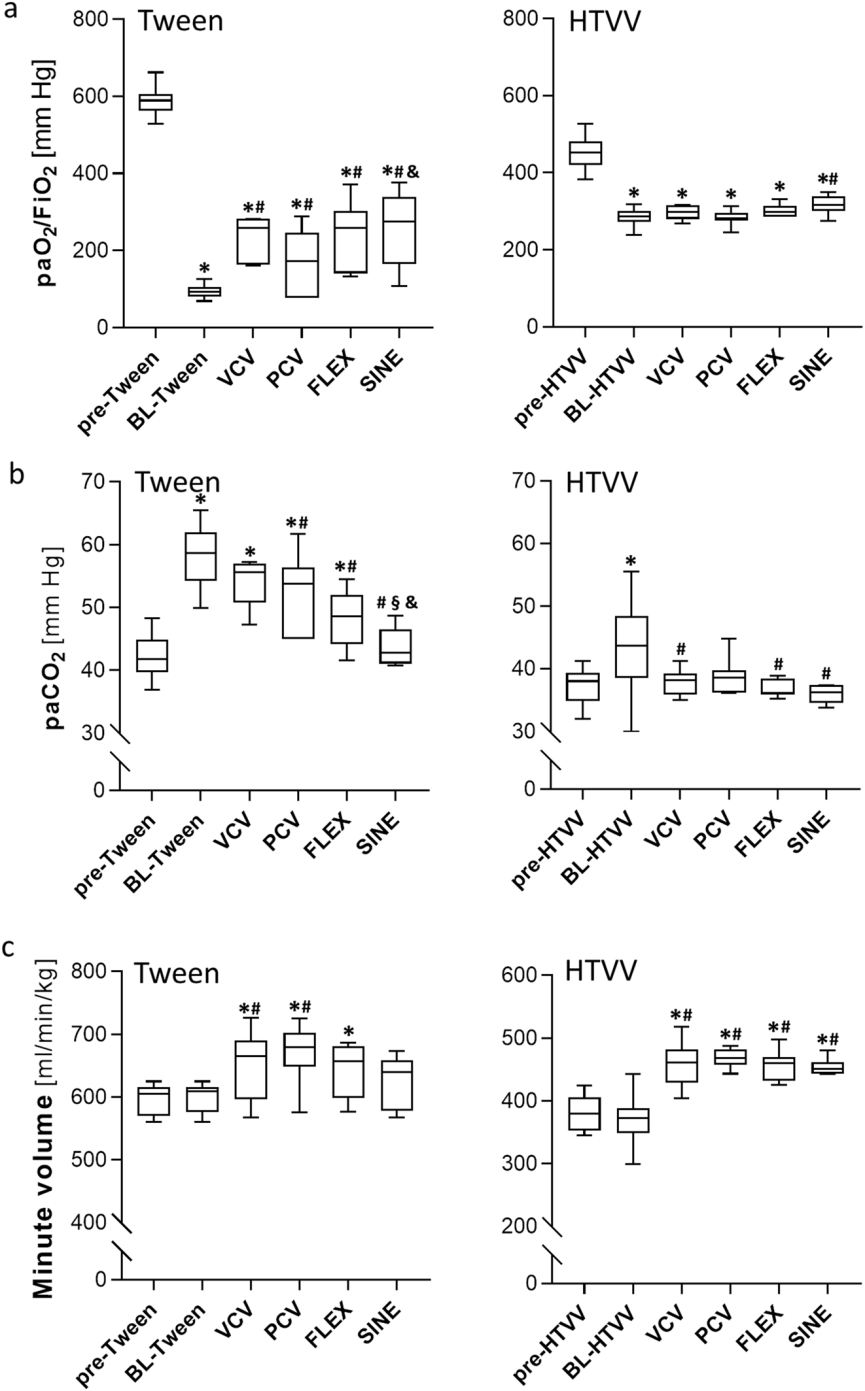
Blood gases and minute volumes. (**a**) Ratio of oxygen partial pressure and inspiratory oxygen fraction (paO_2_/FiO_2_, (**b**) arterial carbon dioxide partial pressure (paCO_2_, and (**c**) weight adapted minute volume for surfactant depletion lung injury model (Tween, left panels) and high tidal volume ventilation lung injury model (HTVV, right panels). Variables are shown before (pre-Tween and pre-HTVV, both n = 28) and after induction of the respective lung injury (BL-Tween and BL-HTVV, both n = 28), and during mechanical ventilation (n = 7/group) with volume controlled ventilation (VCV), pressure controlled ventilation (PCV), VCV + flow-controlled expiration (FLEX) and ventilation mode with a sinusoidal airway pressure profile (SINE), respectively. Box plots represent median, upper/lower quartile and error bars minimal/maximal values. *p* < 0.05 for *compared to pre-Tween/pre-HTVV; ^#^compared to BL-Tween/BL-HTVV; ^§^compared to VCV; ^&^compared to PCV and ^+^compared to FLEX.

**Figure 4:**
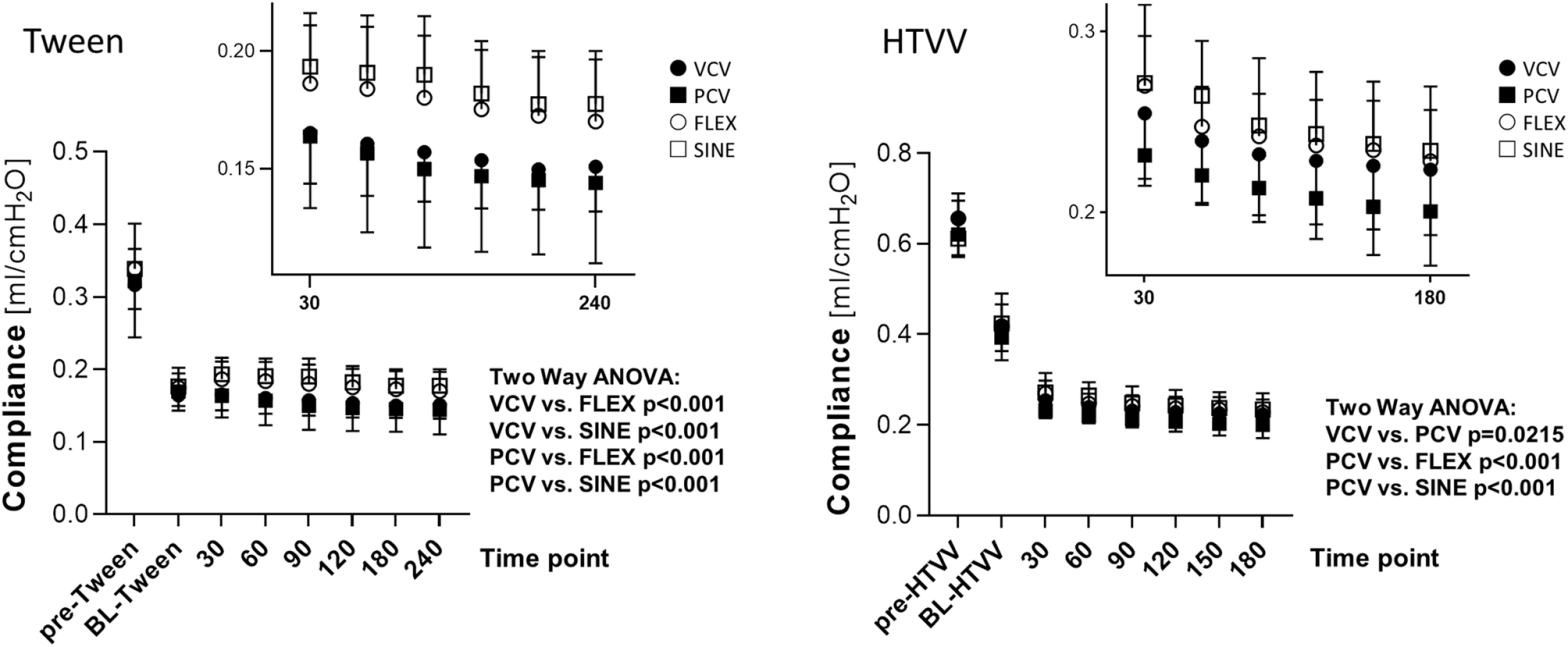
Time course of respiratory system compliances at the beginning of the experiments (pre-Tween and pre-HTVV, both n = 28), after induction of lung injury (BL-Tween and BL-HTVV, both n = 28) and during mechanical ventilation (n = 7/group) with volume controlled ventilation (VCV), pressure controlled ventilation (PCV), VCV + flow-controlled expiration (FLEX) and ventilation mode with a sinusoidal airway pressure profile (SINE), respectively. For facilitation, ventilation mode depending compliance changes during profile specific ventilation in both sets are rescaled and shown as an insert. Data represent mean  ± SD. To void distortion of the statistical analysis due to the considerable compliance drop at onset of lung injury the two-way ANOVA was performed on BL-and subsequent time course data points. P-values show statistical differences between the ventilation profiles.

Induction of Tween or HTVV lung injury (baseline, BL) resulted in significant increase of peak airway pressure (from 10 to 16 cm H_2_O for Tween and from 34 to 36 cmH_2_O for HTVV). The paO_2_/FiO_2_ ratio decreased (from 587 to 95 mmHg for Tween and from 453 to 287 mmHg for HTVV), the average paCO_2_ increased (from 42 to 58 mmHg for Tween and from 37 to 43 mmHg for HTVV) at preserved minute volumes (Fig. 3) and respiratory system compliance (Fig. 4) was reduced by 47% in the Tween set and by 38% in the HTVV set.

### Effect of the ventilation mode on paO_2_, paCO_2_ and minute volume

In the Tween set the paO_2_/FiO_2_-ratio recovered partly with all ventilation modes, thereby, SINE resulted in highest paO_2_/FiO_2_-ratio. In the HTVV set, only animals ventilated with SINE revealed a small but significant increase of the paO_2_/FiO_2_-ratio (Fig. 3a) compared to BL.

In the Tween set, paCO_2_ was significantly lower during ventilation with PCV, FLEX or SINE compared to baseline (Fig. 3b). Additionally, paCO_2_ was significantly lower in animals ventilated with SINE than in animals ventilated with VCV and PCV and was comparable to paCO_2_ before lung injury. In the HTVV set, paCO_2_ remained in the physiological range regardless of the ventilation mode. Thereby, respiratory rate with SINE was lowest in both lung injury models (Supplementary Table ST1A and B). In the Tween set, weight adapted minute volume was higher for animals ventilated with VCV or PCV compared to baseline but not with FLEX or SINE (Fig. 2c). In the HTVV set, applied minute volumes were comparable between all ventilation modes.

### Effect of the ventilation mode on compliance, mean airway pressure and volume

In both sets the changes in the respiratory system compliance after onset of lung injury depended on the ventilation profile (*p* < 0.001). Furthermore, in the HTVV set compliance depended on time (*p* < 0.001, Fig. 4). In the Tween set, ventilation with VCV and with PCV resulted in continuously decreasing compliance. By contrast, ventilation with SINE and FLEX showed initially a small improvement of respiratory system compliance. During further ventilation with SINE and FLEX respiratory system compliance decreased slightly but remained above the compliance after induction of lung injury and remained higher than during VCV and PCV, respectively. In the HTVV set, respiratory system compliance decreased during ventilation with VCV, PCV, FLEX and SINE. However, during ventilation with SINE and FLEX compliance was higher than with VCV and PCV (Fig. 4, HTVV insert).

**Figure 5:**
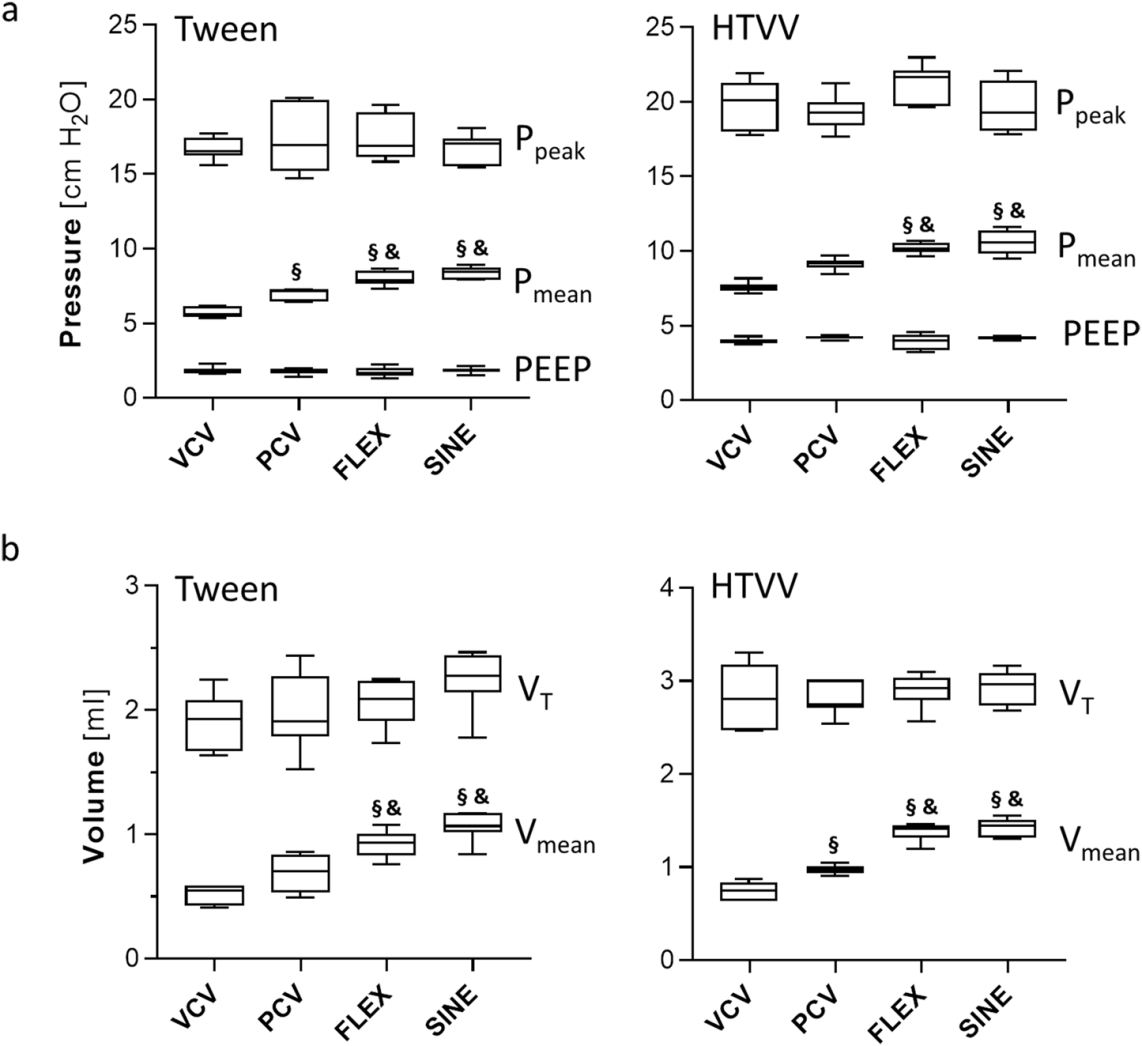
(**a**) Average (arithmetic mean) airway pressure (P_mean_) and (**b**) average (arithmetic mean) volume over time (V_mean_) for mechanical ventilation (n = 7/group) with volume controlled ventilation (VCV), pressure controlled ventilation (PCV), VCV + flow-controlled expiration (FLEX) and ventilation mode with a sinusoidal airway pressure profile (SINE) for the Tween Set (left panels) and the HTVV set (right panels). Box plots represent median, upper/lower quartile and error bars minimal/maximal values. *p* < 0.05 for *compared to pre-Tween/pre-HTVV; ^#^compared to BL-Tween/BL-HTVV; ^§^compared to VCV; ^&^compared to PCV and ^+^compared to FLEX.

Interestingly, at comparable PEEP and peak airway pressure and tidal volumes, the mean airway pressure (Fig. 5a) and the mean of the volume curve of a breathing cycle (Fig. 5b) revealed ventilation mode dependent differences. In the Tween set FLEX and SINE ventilation showed significantly higher mean airway pressure and mean of the volume curves compared to PCV. In the HTVV set the mean of the volume curves of FLEX and SINE, and mean airway pressure of PCV, FLEX and SINE ventilated animals were significantly higher than in VCV-ventilated animals.

### Effect of the ventilation mode on lung injury and inflammation

**Figure 6:**
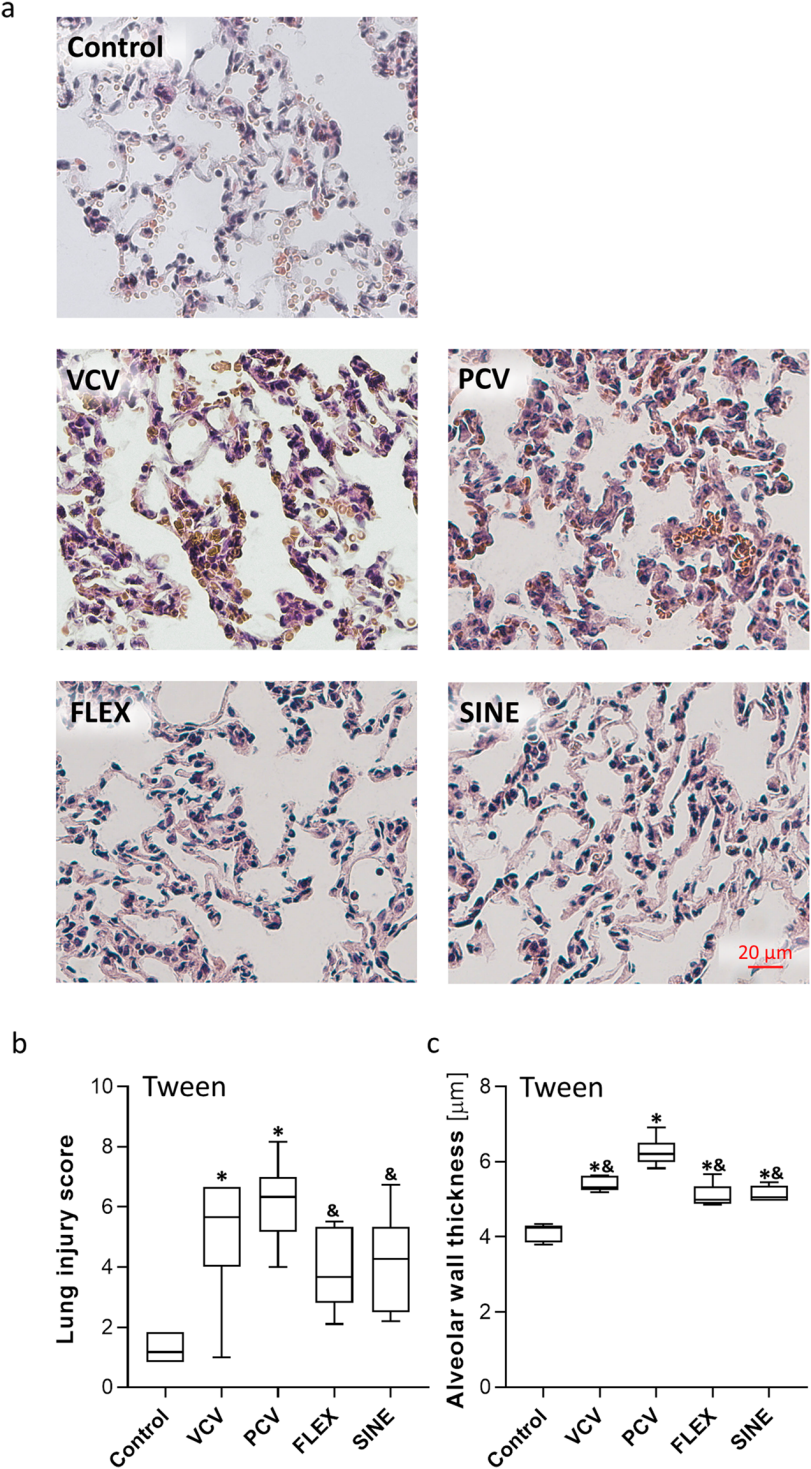
Effect of Tween lavage and ventilation mode on lung injury. (**a**) H&E stained tissue sections and (**b**) lung injury score and (**c**) alveolar wall thickness (n = 5/7 control/ventilation groups, respectively). VCV: volume controlled ventilation; PCV: pressure controlled ventilation; FLEX: VCV + flow-controlled expiration and SINE: ventilation mode with a sinusoidal airway pressure profile. Box plots represent median, upper/lower quartile and error bars minimal/maximal values. *p* < 0.05 for *compared to pre-Tween/pre-HTVV and ^&^compared to PCV.

**Figure 7:**
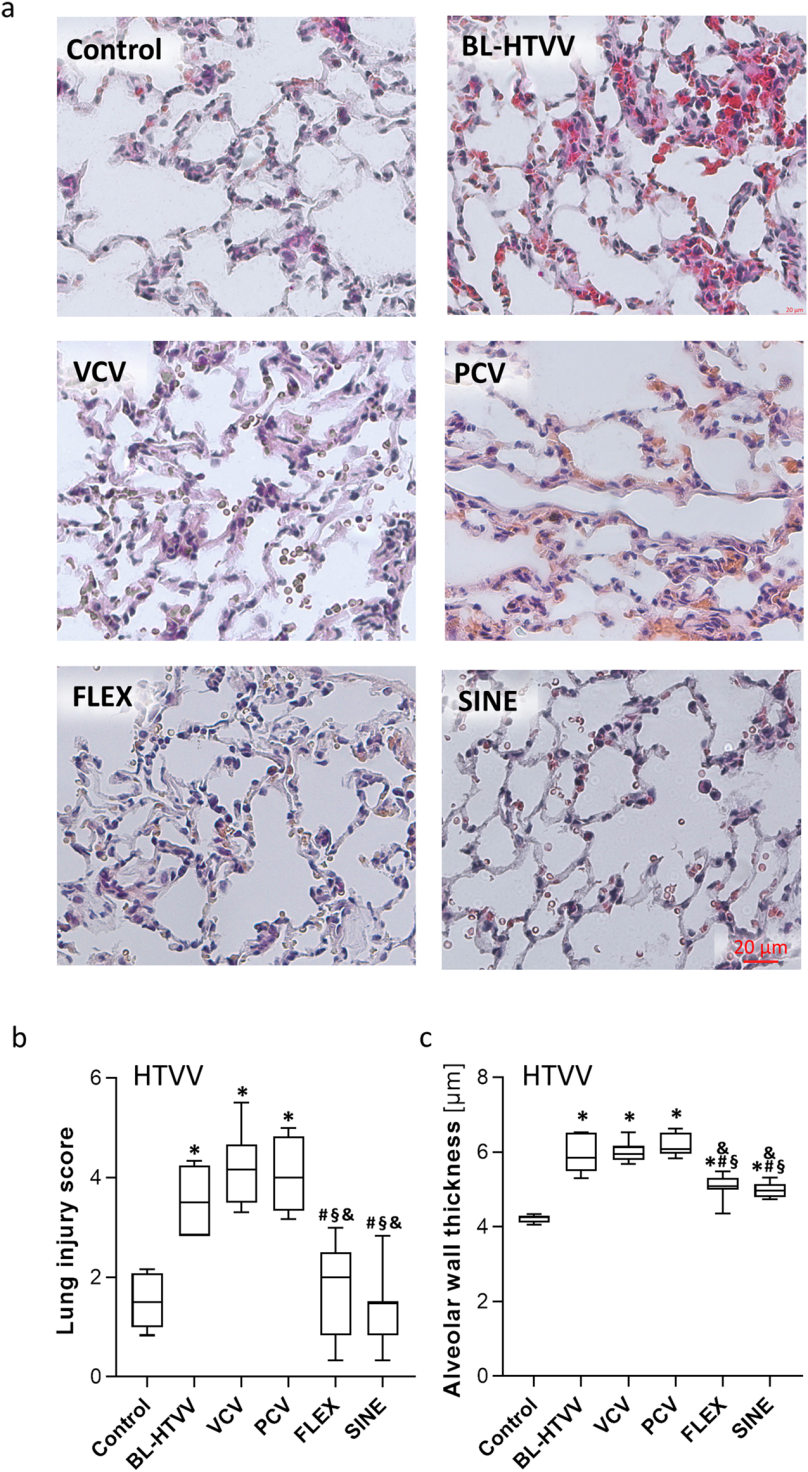
Effect of HTVV and ventilation mode on lung injury. (**a**) H&E stained tissue sections and (**b**) lung injury score and (**c**) Alveolar wall thickness (n = 5/7 control and BL-HTVV/ventilation groups, respectively). VCV: volume controlled ventilation; PCV: pressure controlled ventilation; FLEX: VCV + flow-controlled expiration and SINE: ventilation mode with a sinusoidal airway pressure profile. Box plots represent median, upper/lower quartile and error bars minimal/maximal values. *p* < 0.05 for *compared to pre-Tween/pre-HTVV; ^#^compared to BL-Tween/BL-HTVV; ^§^compared to VCV; ^&^compared to PCV and ^+^compared to FLEX.

In the Tween set, ventilation with VCV, PCV, FLEX or SINE resulted in significant increase of lung injury in terms of thicker alveolar walls, occasional haemorrhages and elevated count of inflammatory infiltrates compared to control animals (Fig. 6). Overall lung injury and particularly alveolar wall thickness were highest after PCV and VCV. In the HTVV set, haemorrhage, infiltrates and alveolar wall thickness were pronounced already after induction of lung injury compared to healthy control (Fig. 7). Subsequent ventilation with VCV and PCV did not induce additional lung injury. By contrast, ventilation with SINE and FLEX resulted in significantly lower alveolar wall thicknesses and lung injury scores compared to VCV and PCV and moreover, compared to the degree at induction of lung injury.

Releases of macrophage inflammatory protein 2, infiltrates (neutrophils and macrophage), total protein in the bronchoalveolar lavage fluid and wet-to-dry ratio of the lung were independent of the ventilation mode (Supplementary Table ST3A and B).

## Discussion

In two separate animal models of lung injury blood gas balance, respiratory system mechanics and lung histology were modulated by the applied ventilation mode suggesting that the pressure course of the ventilation mode may provide additional settings for lung protective ventilation. Thereby, ventilation with SINE improved most efficiently oxygenation (in both sets) and CO_2_ elimination (in the Tween set) at slightly reduced minute volumes in impaired lungs. In today’s clinical practice, a patient’s oxygenation is controlled by the pressure settings (PEEP and/or peak pressure) and CO_2_ elimination via minute volume (controlled via tidal volume and/or respiratory rate). Since PEEP, peak airway pressure and tidal volume settings were equal in all respective groups of both animal sets, we attribute the gas exchange improvements to the airway pressure profile. As a direct result of controlling the pressure course during FLEX and SINE, the expiration and the corresponding airway pressure dynamics appeared reduced (‘decelerated’). Reasoned by this ‘deceleration’, each breathing cycle provides a longer time period (particularly in the expiratory phase) in which the lung is effectively supplied with air. Thus, in spite of identical peak airway pressure and tidal volume, a breathing cycle with FLEX or SINE is characterized by higher mean airway pressure and higher mean of the volume curve compared to VCV and PCV. The increase of mean airway pressure and mean volume over one breathing cycle with FLEX and SINE might be the mechanisms underlying the improved gas exchange. The higher mean airway pressure during ventilation with SINE contributes to a higher O_2_-diffusion gradient from the alveolar space to the blood, and consequently improves oxygenation^10^. The increased time of effective presence of air in the lung increases the functional diffusion area that is in turn related to CO_2_ exchange. Therefore, the observed increase of the mean volume over time could underlay the improved efficiency of CO_2_ elimination during ventilation with FLEX and particularly with SINE. This concept is supported by the slightly improved respiratory system compliance and further by alveolar microscopy video-imaging (Supplementary Video SV1; Supplementary Figure SF1) displaying the time courses of the relative alveolar area. Both, FLEX and SINE provided smoother and prolonged alveolar recruitment compared to VCV and PCV and particularly prevented the tidal alveolar derecruitment that is characteristic for conventional passive expiration^11^. Additionally, the lung injury score and particularly the alveolar wall thickness in the HTVV set, were lower, when animals were ventilated with FLEX and SINE. A thinner alveolar wall thickness is related to a smaller distance of diffusion for gas exchange^12^. Therefore, we assume that reduced alveolar wall thickness during FLEX and SINE ventilation has also contributed to improved gas exchange.

A reduced alveolar wall thickness was also shown in a previous study that investigated respiratory response of flow-controlled expiration compared to VCV in a pig model of acute lung injury^4^. Strikingly, in the HTVV model alveolar lung injury and wall thickness after ventilation with FLEX and SINE was lower compared to alveolar wall thickness after induction of lung injury. Therefore, it could be speculated that a ventilation mode with smoothened profile supports recovery of alveolar walls, which apparently indicates some lung protective potential.

Differences in the lung injury score and alveolar wall thickness cannot be explained with different degrees of induced lung injury since baseline values in respiratory variables and in the inflammatory responses were comparable in all respective groups of the two lung injury models. Indeed, in a recent in vitro study, decelerated dynamics of the strain profile were strongly related to reduced inflammatory response^13^. Those results did not translate to the inflammatory responses in the presented in vivo models of experimental lung injury. First, in contrast to the in vitro study we investigated the effects of the ventilation modes after the lung injury was established and not before or during its recovery. Second, in the in vivo model of experimental lung injury the initially induced level of inflammation may have been beyond the range in which the temporal profile of the ventilation curve influences the cellular response significantly.

The effects of VCV, PCV, FLEX and SINE on the two lung injury models were not intended to be compared directly. However, the results of the presented pre-clinical study revealed lung protective potential of mechanical ventilation with a controlled (e.g. SINE) airway pressure profile in the two independent lung injury models. The reported improvement of paO_2_/FiO_2_-ratio may appear small in regard to clinical practice. However, the lung function is intimately related to the degree of lung injury and for this reason oxygenation is used to define severity of ARDS^14^. To our knowledge, oxygenation is also a basic estimate for taking adequate measures in the critical care unit. Therefore, we assume that our study discloses basic mechanisms and perspective to improve oxygenation, and may provide lung protection by modification of the airway pressure profile. This assumption is supported by other studies employing time control of the ventilation profile (namely based on airway pressure release or high-/multi- frequency mechanical ventilation) which although not based on controlled expiration showed improvements of lung injury and gas exchange on behalf of increased mean airway pressure and maintained lung recruitment^15–17^.

Blood pressure, heart rate and arterial blood gases, which were monitored in our animal models cannot provide an estimate on potential profile-related pulmonary shunt effects, which may play an additional or an important role in the efficiency of gas exchange^12^. Therefore, our approach of mechanical ventilation with a sinusoidal airway pressure curve should be further investigated in other conditions of lung impairment, particularly with respect to chronic diseases. The potential clinical benefits of ventilation with a sinusoidal airway pressure profile (e.g. improved gas exchange, respiratory compliance and lung injury) are to be demonstrated in large animals and in patients. From a technical point of view, modern ventilators would be capable to fully regulate the breathing cycle. Clinical application of a sinusoidal ventilation mode would require only a modification of a ventilator’s control.

Today, lung protective mechanical ventilation includes small tidal volumes^14^ and PEEP guided by individual characteristics of the respiratory system^18^. The insights of the presented study imply that the efficiency of mechanical ventilation can be improved by a modification of the ventilation mode beyond those measures and open a new approach for improved gas exchange and potentially lung protective mechanical ventilation.

Ventilation with SINE improved gas exchange and respiratory system mechanics, and resulted in lower lung injury scores in two models of lung injury. Smoothing the airway pressure profile of mechanical ventilation may be a novel approach for lung protective ventilation beyond low tidal volume and high PEEP strategies.

## Supplementary information

Supplementary information 1.

Supplementary information 2.

## Data Availability

The datasets used and/or analysed during the current study are available from the corresponding author upon reasonable request.
