# Supplementary material for: Sine ventilation in lung injury models: a new perspective for lung protective ventilation
Source: Sci Rep. 2020 Jul 16;10:11690. doi: 10.1038/s41598-020-68614-x (PMC7366701; doi:10.1038/s41598-020-68614-x)
Supplement: Supplementary file 1 — Supplementary information 1. [file 41598_2020_68614_MOESM1_ESM.pdf]

Sine ventilation in lung injury models – a new perspective for lung protective ventilation

Sashko Spassov[1]*, Christin Wenzel[1], Sara Lozano–Zahonero, Dimona Boycheva, Lea Streicher, Johannes Schmidt, Stefan Schumann

Department of Anesthesiology and Critical Care, Medical Center – University of Freiburg, Faculty of Medicine, University of Freiburg, Freiburg, Germany

[1]both authors have contributed equally

*Corresponding author:

Workgroup Clinical Respiratory Physiology

Department of Anesthesiology and Critical Care,

Medical Center – University of Freiburg,

Hugstetter Str. 55,

79106 Freiburg, Germany

Phone: +49 76127023080

Fax +49 76127023960

E–mail: sashko.spassov@uniklinik–freiburg.de

Supplementary table ST1 Respiratory parameters of VCV, PCV, FLEX and SINE related to pre–injury and BL–injury timepoints in A) the surfactant washout set (Tween) and B) in the high tidal volume ventilation set (HTVV).

A) Tween set	Pre–Tween				BL–Tween			
Variables	VCV	PCV	FLEX	SINE	VCV	PCV	FLEX	SINE
**paO_2/FiO_2** [mmHg]	586±48	584±31	592±20	585±20	90±15	90±13	96±11	104±16
**$paCO_2$** [mmHg]	41±3	42±4	43±3	42±2	59±4	58±5	59±5	58±3
**Minute volume** [ml/min/kg]	592±27	599±19	599±20	599±23	592±27	606±15	599±20	599±23
**Compliance** [ml/cmH_2O]	0.32±0.03	0.32±0.08	0.34±0.06	0.34±0.03	0.16±0.01	0.17±0.03	0.17±0.03	0.18±0.02
**B) HTVV set**	**Pre–HTVV**				**BL–HTVV**			
**Variables**	**VCV**	**PCV**	**FLEX**	**SINE**	**VCV**	**PCV**	**FLEX**	**SINE**
**paO_2/FiO_2** [mmHg]	448±47	444±36	447±22	471±25	295±17	291±18	268±18	292±15
**$paCO_2$** [mmHg]	37±3	38±4	37±3	37±2	41±7	45±4	44±6	44±9
**Minute volume** [ml/min/kg]	384±32	376±24	286±24	363±24	360±22	386±27	361±34	372±43
**Compliance** [ml/cmH_2O]	0.66±0.08	0.62±0.05	0.66±0.06	0.61±0.08	0.41±0.07	0.39±0.03	0.41±0.05	0.42±0.06

All animals were ventilated with volume controlled ventilation (VCV) and subsequently randomised to one of the four ventilation mode VCV, pressure controlled ventilation (PCV), VCV+flow–controlled expiration (FLEX) and ventilation with a sine pressure profile (SINE), (n=7/group). All variables were comparable between the groups for each pre–injury and BL–injury timepoints, respectively. Data represent mean ± SD. ANOVA followed by Tukey post–hoc test. paO2/FiO2: ratio of arterial partial pressure of oxygen; paCO2: arterial partial pressure of carbon dioxide.

Supplementary table ST2 Experimental and physiological variables.

A) Tween set			Ventilation mode			
**Variables**	**pre-Tween**	**BL-Tween**	**VCV**	**PCV**	**FLEX**	**SINE**
**weight [g]**	–	–	279±23	287±33	299±28	311±30
**body temperature [°C]**	36.7±0.7	36.5±0.5	37.0±0.1	36.8±0.2	36.9±0.2	36.9±0.2
**heart rate [1/min]**	300±26	278±24*	303±17	292±18	286±14	284±16
**mBP [mmHg]**	146±8	125±20*	101±8*#	103±10*#	101±8*#	102±5*#
**respiratory rate [1/min]**	74±3	75±3	82±7*#	84±7*#	80±6*#	78±6
**peak pressure [cmH_2O]**	10.4±1.2	16.4±1.4*	16.9±0.7*	17.5±2.1*	17.5±1.5*	16.9±1.0*
**blood pH**	7.46±0.06	7.33±0.03*	7.35±0.03*	7.36±0.02*#	7.37±0.02*#	7.41±0.04*#§&+
**blood bicarbonate [mmol/l]**	28±1.0	26±1.0*	26±1.3*	26±0.6*	25±1.0*	26±1.5*
**base excess [mmol/l]**	5±1.5	4±1.4*	3±1.9*	3±1.2*	2±1.6*#	3±1.8*
**haematocrit [vol%]**	42±1.5	41±1.51	38±1.9*#	38±1.9*#	37±1.8*#	39±1.5*
**haemoglobin [g/dl]**	14.9±0.9	14.4±0.7	13.7±0.9*	13.9±0.9*	13.2±1.2*#	14.4±0.5

B) HTVV set						
**Variables**	**pre-HTVV**	**BL-HTVV**	**VCV**	**PCV**	**FLEX**	**SINE**
**weight [g]**	–	–	402±34	391±26	400±26	393±20
**body temperature [°C]**	36.2±0.7	36.8±0.4	36.5±0.1	36.7±0.2	36.6±0.3	36.8±0.3
**heart rate [1/min]**	284±23	271±21	292±24	297±23	292±20	292±25
**mBP [mmHg]**	133±17	78±8*	97±5*#	89±9*	87±7*	88±7*
**respiratory rate [1/min]**	14±0	14±1	67±3*#	67±2*#	65±3*#	62±1*#&§+
**peak pressure [cmH_2O]**	33.9±1.9	35.8±1.4*	19.8±1.6*#	19.3±1.1*#	21.1±1.4*#	19.6±1.6*#
**blood pH**	7.48±0.03	7.39±0.04*	7.42±0.04*	7.41±0.03*	7.43±0.02*	7.43±0.01*
**blood bicarbonate [mmol/l]**	27±0.9	25±1.2*	24±2.0*	24±0.8*	23±1.7*	24±1.1*
**base excess [mmol/l]**	3±1.3	1±2.0*	0±2.3*	0±0.9*	-1±1.0*	-1±1.5*
**haematocrit [vol%]**	43±2	42±2	40±2*	40±2*	40±4*	40±2*
**haemoglobin [g/dl]**	15±1	15±1	15±1	14±1	15±1	14±1

Values are shown for the beginning of the experiment (pre–Tween/pre–HTVV n=28), after induction of lung injury (BL–Tween/ BL–HTVV n=28), and after mechanical ventilation with volume controlled ventilation (VCV, n=7), pressure controlled ventilation (PCV, n=7), VCV+flow–controlled expiration (FLEX, n=7) and ventilation mode with a sinusoidal airway pressure profile (SINE, n=7). Data represent mean ± SD. ANOVA followed by Tukey post–hoc test: *: p <0.05 vs. pre–Tween/pre–HTVV; #: p <0.05 vs. BL–Tween/BL–HTVV; §: compared to VCV; &: compared to PCV and +: compared to FLEX.

mBP: mean blood pressure; PEEP: positive end–expiratory pressure. HTVV: high tidal volume ventilation; mBP: mean blood pressure; PEEP: positive end–expiratory pressure

Supplementary table ST3 Effect of the ventilation mode on inflammation in the Tween set A) and in the HTVV set B).

**A) Tween set**	**Ventilation mode**			
**Variables**	**VCV**	**PCV**	**FLEX**	**SINE**
**MIP–2/total protein [pg/µg]**	0.14±0.06	0.14±0.09	0.16±0.05	0.13±0.09
**neutrophils in BALF [%]**	57.5±7.9	57.7±5.7	58.5±4.4	54.8±5.8
**macrophage in BALF [1 x10^5/ml]**	8.5±3.0	10.6±7.2	9.5±4.7	7.7±2.1
**lung W/D ratio**	5.7±0.8	5.9±0.8	5.8±1.0	5.4±0.4
**total protein in BALF [mg]**	1.2±0.2	1.6±0.6	1.5±0.8	1.3±0.4

**B) HTVV set** **Variables**	**VCV**	**PCV**	**FLEX**	**SINE**
**MIP–2/total protein [pg/µg]**	0.15±0.08	0.09±0.04	0.09±0.05	0.08±0.02
**neutrophils in BALF [%]**	47.6±5.2	48.9±6.0	44.87±8.1	44.2±7.4
**macrophage in BALF [1 x10^5/ml]**	10.0± 3.7	10.5±5.7	9.3±6.8	8.6±2.3
**lung W/D ratio**	8.1±0.8	8.7±0.8	8.7±0.9	8.3±0.8
**total protein in BALF [mg]**	5.0±0.9	5.0±1.3	4.7±1.6	5.1±1.4

Values are shown after mechanical ventilation with volume controlled ventilation (VCV), pressure controlled ventilation (PCV), VCV+flow–controlled expiration (FLEX) and ventilation with a sine pressure profile (SINE), (n=7/group). Data represent mean ± SD. ANOVA followed by Tukey post–hoc test.
BALF: bronchoalveolar fluid; MIP–2: macrophage inflammatory protein 2. HTVV: high tidal volume ventilation;

Supplementary Figure SF1

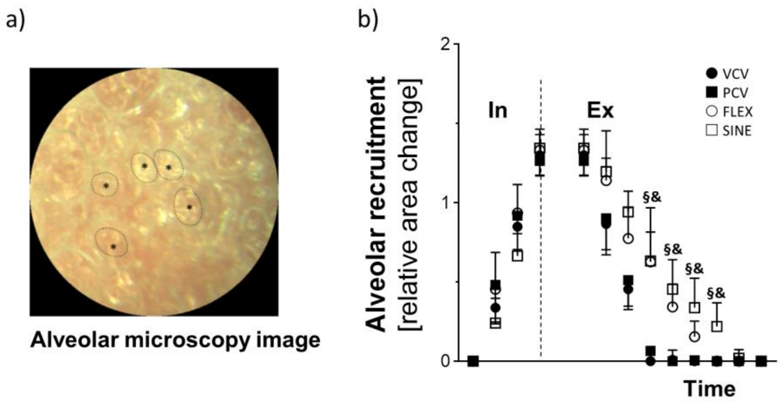

Supplementary Figure SF1 Alveolar microscopy imaging. a) Representative alveolar microscopy image. Asterisks designate subpleural alveoli selected for subsequent analysis of the effect of the ventilation profiles on the time dependent relative alveolar area changes during inspiration and expiration. b) The relative area changes were calculated from subpleural alveolar microscopy imaging (supplementary video S1). Alveoli were defined by, an intense yellow/orange contour of the inner rim and only structures which were presented during the entire experiment were evaluated for size changes. The minimum alveolar size was set to 0. Alveolar microscopy was performed ex vivo on ‘healthy’ and exsanguinated animal as previously described (Schwenninger D. et al. Crit Care Med 2013; 4: 1286–1295). To ensure sufficient time resolution for video documentation of the alveolar dynamics microscopy was performed *ex vivo* applying tidal volume of 10 ml/kg and respiratory rate of 44 breaths / minute and PEEP of 5 cmH_2O. Each of the four ventilation modes were repeated three times changing the order of profiles randomly. Data represent mean + or - SD. Time profile of ventilation mode as well as ventilation mode were significant factors (all $p < 0.0001$, repeated measure two–way ANOVA with Tukey post hoc test). $p < 0.05$ for §: compared to VCV; &: compared to PCV. VCV: volume controlled ventilation, PCV: pressure controlled ventilation, FLEX: VCV+flow–controlled expiration and SINE: ventilation with sine pressure profile.
